# Neuroblastic Tumors of the Adrenal Gland in Elderly Patients: A Case Report and Review of the Literature

**DOI:** 10.3389/fped.2022.869518

**Published:** 2022-05-17

**Authors:** Philip Deslarzes, Reza Djafarrian, Maurice Matter, Stefano La Rosa, Carole Gengler, Maja Beck-Popovic, Tobias Zingg

**Affiliations:** ^1^Department of Visceral Surgery, Lausanne University Hospital, Centre Hospitalier Universitaire Vaudois (CHUV), Lausanne, Switzerland; ^2^Department of Pathology, Institute of Pathology, Lausanne University Hospital, Lausanne, Switzerland; ^3^Department “Woman-Mother-Child”, Lausanne University Hospital, Lausanne, Switzerland

**Keywords:** neuroblastic tumors, ganglioneuroblastoma, elderly patient, preoperative diagnosis, surgical and medical therapy, staging, prognosis, follow up

## Abstract

**Background:**

Neuroblastic neoplasms (NN) include ganglioneuromas (GN), ganglioneuroblastomas (GNB), and neuroblastomas (NB). They generally arise in childhood from primitive sympathetic ganglion cells. Their incidence in adults, especially among elderly, is extremely low.

**Case Presentation:**

This is the case of a 74-year-old woman with history of abdominal pain, weakness and night sweating since several months. Blood pressure was normal. CT-scan showed a 10 cm left adrenal mass, without other pathologic findings. An open left-sided adrenalectomy was performed. Recovery was uneventful with hospital length of stay of 8 days. Based on morphological, immunohistochemical, and molecular features the diagnosis was a nodular GNB. A positron emission tomography (PET) performed 6 weeks after the resection did not show any residual tumor or distant metastases. The patient was followed-up with annual clinical and radiological exams.

**Conclusion:**

This case presentation, associated with a review of the literature, illustrates the importance to include NN in the preoperative differential diagnosis of adrenal tumors in adults and highlights the need for multidisciplinary patient work-up and management.

## Background

Neuroblastic neoplasms (NN) are a heterogeneous group of tumors deriving from the sympatho-adrenal lineage of the neural crest forming the peripheral sympathetic nervous system. They include a wide spectrum of neoplasms with various degrees of cellular maturation and biological aggressiveness (1–4). Ganglioneuromas (GN) show an indolent behavior with benign clinical course, while, on the other hand, neuroblastomas (NB) are malignant tumors with a much worse prognosis. Between GN and NB, ganglioneuroblastomas (GNB) exhibit some intermediate malignant potential depending on the tumor subtype (intermixed or nodular) and on other specific criteria (3).

NB are the most frequent extra cranial solid tumors in early childhood, (8–10% of childhood cancers), while GNB are very rare, including in the pediatric population (0.5 cases/100,000 children) (4–7). In adults and elderly (>65 years) population, the incidence of NB and GNB is even rarer (0.01 cases /100,000) (8, 9). More than 97% of cases occur in patients younger than 10 years-old and the median age at diagnosis is 2 years (10). The most common sites of origin of NB and GNB are the adrenal glands (40%) and extra-adrenal retroperitoneum (25%), although they can arise anywhere throughout the sympathetic nervous system (neck, chest, pelvis) (11, 12). One of the most important prognostic factors is age at diagnosis. Of note, prognosis of NB in elderly is generally poor (13). Elderly people with NB have a significantly worse outcome than children younger than 5 years (14–16).

Because of the rarity of NN in adults, the natural history in this population is unknown and there are no established treatment guidelines. To the best of our knowledge, no case of adrenal GNB in elderly patients has been reported in literature until now (Table 1). In the present paper, we discuss the clinico-pathological features, work-up and management of an adrenal GNB diagnosed in a 74-year-old patient.

**Table 1:** Adrenal neuroblastic neoplasms in elderly patients (>65 years) in the English literature from 1976 to 2021.

**Case**	**Age at Dx (years)**	**Sex**	**Stageat Dx**	**Symptoms**	**Lateralization**	**Initial therapy**	**Final pathology**	**Follow-up** **(years)**	**Outcome**	**Urine CCh**	**References**
1	65	F	NA	Headache, malaise, facial flushing	R	Surgery	GN	0.5	Alive	Neg	(17)
2	67	F	NA	Asymptomatic (microscopic hematuria)	L	Surgery	GN	0.5	Alive	Neg	(18)
3	75	F	IV	Bilateral lower extremity weakness, numbness	L	Surgery	NB	2.2	Dead	Neg	(19)
4	74	F	I	Abdominal pain, weakness, night sweating	L	Surgery	GNB	0.75	Alive	NA	

*Dx, diagnosis; R, right; L, left; CCh, catecholamines; NB, Neuroblastoma; GN, Ganglioneuroma; NA, not available*.

## Case Presentation

**Figure 1:**
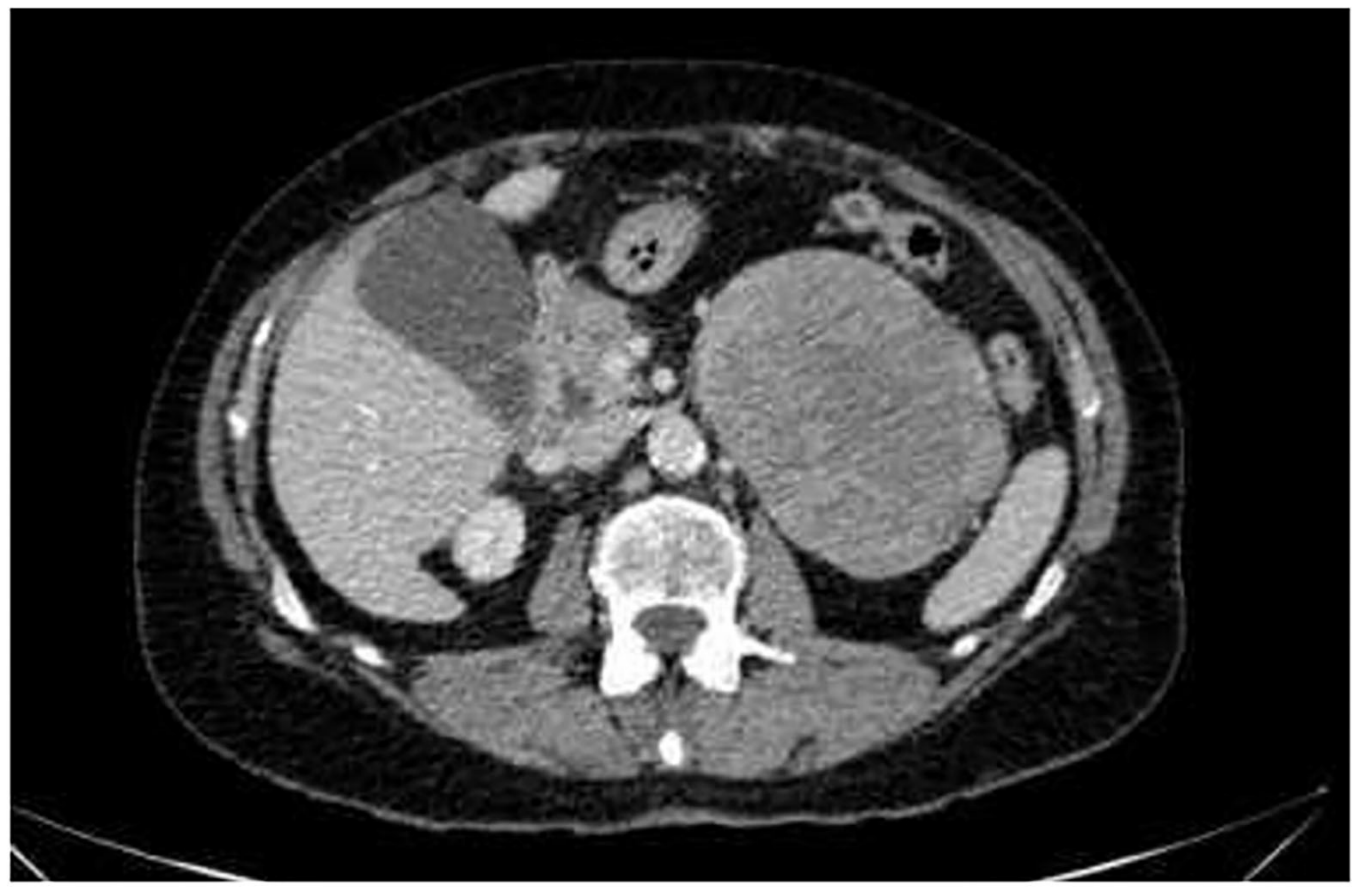
CT-scan of the abdomen. Axial CT scan of the abdomen reveals a large abdominal mass measuring 10 × 8 × 10 cm without lymphadenopathy.

**Figure 2:**
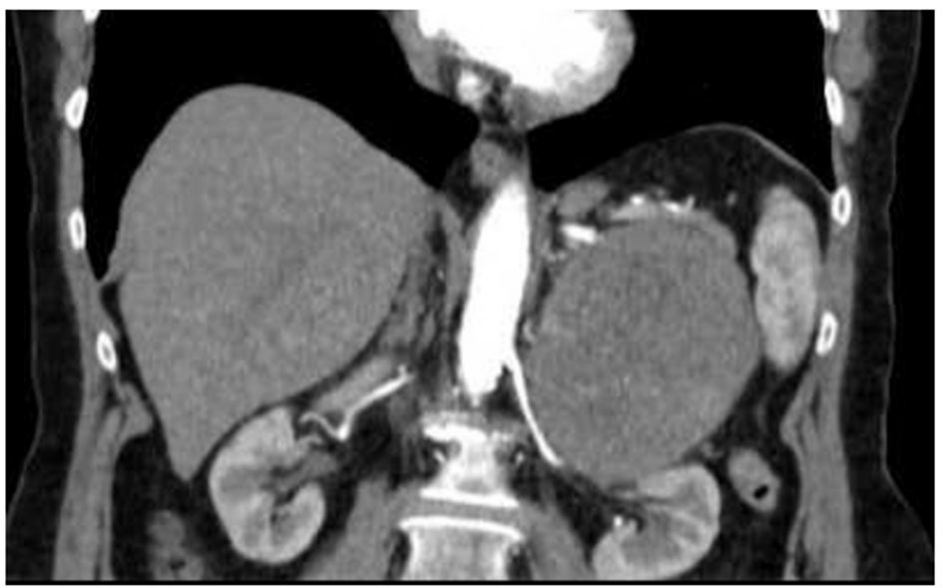
CT-scan of the abdomen. Coronal-view CT scan of the abdomen reveals a large abdominal mass measuring 10 × 8 × 10 cm without lymphadenopathy.

A 74-year-old woman consulted her GP with history of abdominal pain, weakness and night sweating for months. She was on prednisone and pregabalin for fibromyalgia. The patient had no history of hypertension. The physical examination showed a palpable mass in the left upper quadrant, blood pressure was normal. A thoraco-abdominal contrast-enhanced CT-scan, showed a 10 × 10 × 8 cm mass at the upper pole of the left kidney (Figures 1, 2) and no other suspicious lesions. A CT-guided core needle biopsy of the mass was performed with histology findings compatible with a possible adrenocortical tumor (immunohistochemistry was positive for vimentine, racemase and synaptophysin, but was negative for S-100 and chromogranin).

Serum catecholamines showed elevated nor-metanephrine (9.72 nmol/l, N: 0.04–1.39) and methoxytyramine (0.62 nmol/l, N: <0.06), but normal metanephrine levels (high negative predictive value for excluding pheochromocytoma) (17). Urine catecholamines were not evaluated. The other laboratory work-up showed normal cortisol, androstenedione, DHEA and testosterone levels.

**Figure 3:**
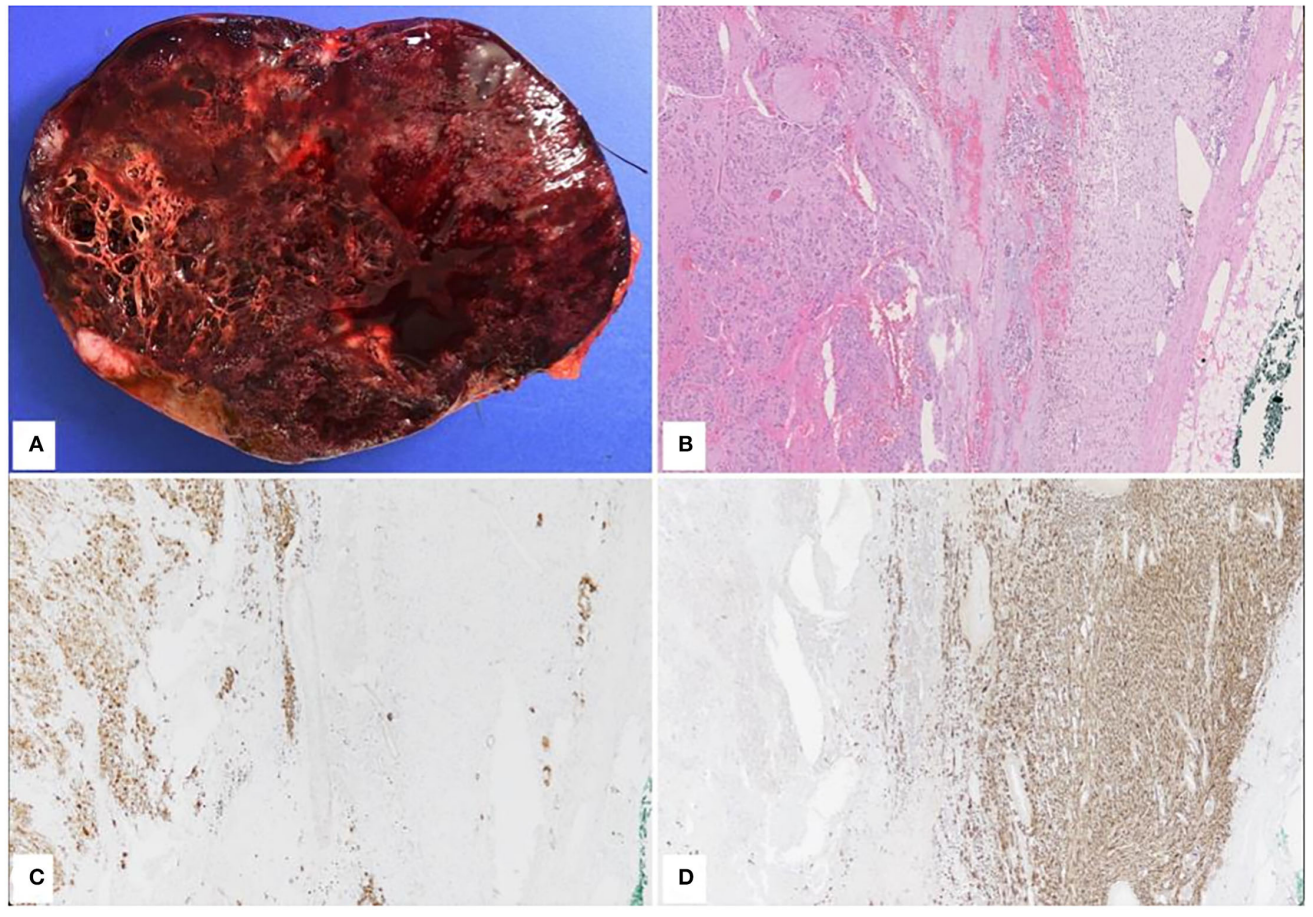
Pathological features. Macroscopically, The cut surface was heterogeneous with solid and microcystic features, hemorrhagic and necrotic areas, and some white nodules. The tumor was well-delimitated showing solid and microcystic features with hemorrhagic and necrotic areas. **(A)** Histologically, the tumor showed two main components. The central portion of the tumor was composed of ganglion cells at different stage of maturation, embedded in a fibrillary network **(B)** left portion of the image. The ganglion cells were positive for synaptophysin, chromogranin and MAP2 while they were negative for SF1, inhibin, MYC, CD99, cytokeratin, GFAP, and ATRX. SDHB was expressed. **(C)** The second component of the tumor was located at the periphery and included spindle Schwann cells positive for S100 **(D)**.

An open left adrenalectomy was performed through a left subcostal laparotomy. The definitive diagnosis was nodular GNB (Figure 3).

Recovery was uneventful with hospital stay of 8 days. Following our multidisciplinary endocrinology board, the staging was completed with PET-CT, which showed no distant lesions. It was programmed a clinical follow up at 1 month after the surgery with a favorable clinical course and a resolution of all symptoms. The patient was followed with annual clinical exams and CT-scans.

## Discussion

### Diagnosis

The clinical presentation of NN is very heterogeneous and depends on the site of origin and the presence of metastatic disease (18). Approximately two thirds of NN are localized in the abdomen and two thirds of the abdominal NN originate from the adrenal glands (11, 12). However, it is worth noting that they can arise anywhere throughout the sympathetic nervous system, such as the thorax or the neck (11).

NB and GNB can secrete catecholamines, and their derivates such as vanillylmandelic acid and homovanillic acid. Determination of catecholamine levels and their serum metabolites is important for the initial screening, diagnosis, differential diagnosis (i.e., pheochromocytoma vs. adrenocortical carcinoma), and for the evaluation of treatment efficacy (19). Neuroblastoma cells may release ferritin and patients with high ferritin level or increased levels of lactate dehydrogenase tend to have a worse prognosis (20).

According to a recent retrospective study, 48% of patients with abdominal NB and 29% of patients with abdominal GNB had metastatic disease (18). Thus, functional imaging like I_123_-MIBG-scan or FDG PET-CT, are essential in the assessment of NB and GNB to define the extent of disease at diagnosis and to follow the treatment response (21, 22). Notably, it was shown that FDG can be useful when interpretation of MIBG is unclear (23). Some studies have shown that Ga_68_-DOTATATE PET-CT has a better sensitivity, quicker clearance, limited toxicity and less radiation exposure than _123_I-MIBG and could be used for staging of NN (24–26). Due to the rarity of intracranial metastases (0.7% in stage IV disease in children), brain CT or MRI should be only performed if intracranial involvement is clinically suspected (27, 28). The final diagnosis has to be confirmed by histology.

### Staging and Prognosis

GNB, NB and GN are three separate diseases with different outcomes: malignant for NB, intermediate for GNB and benign for GN. NB and GNB have different clinical behaviors with some tumors showing spontaneous regression and others progressing to metastatic dissemination (27).

There are currently two classification systems. The International Neuroblastoma Staging System (INSS) and the International Neuroblastoma Risk Group Staging System (INRGSS), are summarized in Tables 2, 3 (30). The risk stratification is based on age at diagnosis, INRG tumor stage, grade of tumor differentiation, histologic category, DNA ploidy, and number of copies at the MYCN oncogene locus and at chromosome 11q (29).

**Table 2:** INRG Tumor staging system (29).

**Stage L1**	**Localized tumor not involving vital structures, as defined by the list of IDRFs, and confined to one body compartment**
**Stage L2**	**Local-regional tumor with presence of one or more IDRFs**
**Stage M**	**Distant metastatic disease**
**Stage MS**	**Metastatic disease in children younger than 18 months, with metastases confined to liver and/or bone marrow**

*IDRFs, Image-defined risk factors*.

**Table 3:** INSS Tumor staging system (29).

**Stage 1**	**Localized tumor with complete excision, with or without microscopic residual disease; representative ipsilateral lymph nodes negative for tumor microscopically. Nodes attached to and removed with the primary tumor may be positive**
**Stage 2A**	**Localized tumor with incomplete gross excision, representative ispsilateral nonadherent lymph nodes negative for tumor microscopically**
**Stage 2B**	**Localized tumor with or without complete gross excision, with ipsilateral non-adherent lymph nodes positive for tumor, enlarged contralateral lymph nodes negative microscopically**
**Stage 3**	**Unrespectable unilateral tumor infiltrating across the midline (beyond the opposite side of the vertebral column) with or without regional lymph node involvement, or midline tumor with bilateral extension via infiltration (unrespectable) or lymph node involvement**
**Stage 4**	**Any primary tumor with dissemination to distant lymph nodes, bone, bone marrow, liver, skin, and/or other organs (except as defined for stage 4S disease)**
**Stage 4S**	**Localized primary tumor (as defined for stage 1, 2A, or 2B disease) with dissemination limited to skin, liver, and/or bone marrow (limited to infants younger than 1 year, marrow involvement or less 10% of total nucleated cells, and MIBG scan findings negative in the marrow**

According to the INRGSS and INSS, our patient qualifies as a stage L1 and a stage 1, respectively. Adults with L1 disease experience an OS of 94, 90, and 69% at 3, 5, and 10 years, respectively (19). The most important prognostic factor is age, but in adults and elderly population data are limited. According to few case reports, the prognosis in adults is worse than among children, because of unfavorable histological characteristics (8, 9, 12, 19). In a retrospective study, Conter et al. observed an OS of adult patients of 18.1 years, 9.8 years and 1.6 years for stage L1, L2 and M, respectively (19).

NN patients with a high quantity of neurotrophin receptors, found on the surface of normal nerve cells and some neuroblastoma cells, especially the nerve growth factor receptor TrkA, may have a better prognosis (20).

In older children, but not in infants and adults, MYCN amplification represents a negative prognostic factor (31). The amplification for MYCN was negative in our patient, strengthening our multidisciplinary board decision for a clinical/radiological follow-up. MYCN amplification occurs in about 40% of high-risk tumors (32).

Outcomes of NN in adults are generally poor, because half of the patients have a stage IV NB at the time of diagnosis (11, 13). 45% of NB are classified as high risk (33).

### Treatment in Children

In children, treatment of NB and GNB is well-defined but remains demanding. Surgical resection is the mainstay for low-risk pediatric cases, whereas high-dose chemotherapy with stem cell rescue, radiation therapy and immunotherapy are indicated in high-risk patients (20). In children with INSS stage 4 disease, intensive cytotoxic chemotherapy offered a better progression-free survival than standard chemotherapy (31). In high-risk tumors (50% of pediatric NB and GNB), multiagent chemotherapy, surgical resection, radiotherapy, myeloablative chemotherapy and autologous stem cell rescue, as well as immunotherapy (i.e., anti-GD2 immunoglobulins, ALK-inhibition molecules) can be used (34).

### Treatment in Adults

Regarding treatment, the presented patient had surgery without chemotherapy or radiation therapy. In adults, due to rarity, there are no standardized guidelines or treatment protocols for NN (22). According to the three case reports mentioned above, the treatment of the GN and NB was surgical without adjuvant treatment. Treatment guidelines usually follow those existing for children or those from rare retrospective studies (4, 14, 20). For adult patients with INRG stage L1 NB, surgery combined with adjuvant radiotherapy offer a better OS than surgical resection alone (20). In the same study, chemotherapy did not show any additional benefit for stages L1 and L2. In high-risk patients, high-dose chemotherapy with hematopoietic stem cell rescue did not show the same benefit when compared to pediatric patients (20). According to pediatric guidelines, surgery alone should be performed for low-risk tumors, while multiagent chemotherapy is effective in patients with relapsing low-risk tumors or in those with intermediate-risk tumors (33). The treatment of choice for metastatic patients is chemotherapy (22).

### Follow-Up

Follow-up with urinary catecholamine levels and imaging is necessary for all pediatric patients treated for NB and GNB (35). As functional tumors are rare in adults, it was decided not to follow-up the urinary catecholamine level in our patient (12).

There are no clear guidelines concerning imaging-based follow-up, but the frequency of visits and duration of observation period depend on age, stage and localization of the tumor. In some children, ultrasound may be used for surveillance, while CT, MRI or even MIBG-scan can be necessary for adults or advanced-stage metastatic disease (34, 36). FDG PET-CT is not needed when a MIBG-scan has been performed, however some NN cells do not absorb MIBG (36). Due to paucity of NB, each case should be discussed in the setting of a multidisciplinary board.

## Conclusion

GNB should be included in the preoperative differential diagnosis of adrenal tumors in adults. In the absence of specific guidelines, management of adrenal NN in elderly is surgical following pediatric protocols and retrospective observational studies.

## Data Availability Statement

The original contributions presented in the study are included in the article/supplementary material, further inquiries can be directed to the corresponding author/s.

## Ethics Statement

Written informed consent was obtained from the individual(s) for the publication of any potentially identifiable images or data included in this article.

## Author Contributions

PD wrote the manuscript and the abstract. RD was the first corrector. MM and TZ were the second correctors. TZ was the coordinator. SL and CG added the pathology section and the pictures. MB-P added some informations concerning her medical specialties. All authors read and approved the final manuscript.

## Funding

Open access funding was provided by the University of Lausanne.

## Conflict of Interest

The authors declare that the research was conducted in the absence of any commercial or financial relationships that could be construed as a potential conflict of interest.

## Publisher's Note

All claims expressed in this article are solely those of the authors and do not necessarily represent those of their affiliated organizations, or those of the publisher, the editors and the reviewers. Any product that may be evaluated in this article, or claim that may be made by its manufacturer, is not guaranteed or endorsed by the publisher.

## Abbreviations

ATRX: Alpha-Thalassemia Mental Retardation-X
DHEA: Dehydroepiandrosterone
CD99: Cluster of Differentiation–99
FDG: Fluorodeoxyglucose
GN: Ganglioneuromas
GNB: Ganglioneuroblastomas
GP: General Practician
GFAP: Glial Fibrillary Acidic Protein
MIBG: Metaiodobenzylguanidine
INSS: International Neuroblastoma Staging System
INRGSS: International Neuroblastoma Risk Group Staging System
NN: Neuroblastic Neoplasms
Neuroblastomas: NB
MAP-2: Microtubule Associated Protein 2
MYC: Myelocytomatosis oncogene
OS: Overall Survival
PET: Positron Emission Tomography
SF1: Splicing Factor 1
SDHB: Succinate Dehydrogenase.
