# Erratum: Neuroblastic tumors of the adrenal gland in elderly patients: A case report and review of the literature

**DOI:** 10.3389/fped.2023.1185364

**Published:** 2023-03-28

**Authors:** Frontiers Production Office

**Affiliations:** Frontiers Media SA, Lausanne, Switzerland

**Keywords:** neuroblastic tumors, ganglioneuroblastoma, elderly patient, preoperative diagnosis, surgical and medical therapy, staging, prognosis, follow up

An Erratum on Neuroblastic tumors of the adrenal gland in elderly patients: A case report and review of the literature By Deslarzes P, Djafarrian R, Matter M, La Rosa S, Gengler C, Beck-Popovic M and Zingg T. (2022) Front. Pediatr. 10:869518. doi: 10.3389/fped.2022.869518

An omission to the funding section of the original article was made in error. The following sentence has been added: “Open access funding was provided by the University of Lausanne”.

The original version of this article has been updated.

